# The Pitfalls of *in vivo* Cardiac Physiology in Genetically Modified Mice – Lessons Learnt the Hard Way in the Creatine Kinase System

**DOI:** 10.3389/fphys.2021.685064

**Published:** 2021-05-14

**Authors:** Craig A. Lygate

**Affiliations:** Division of Cardiovascular Medicine, Radcliffe Department of Medicine, Wellcome Centre for Human Genetics, University of Oxford, Oxford, United Kingdom

**Keywords:** integrative physiology, transgenic, heart failure, creatine kinase, metabolism

## Abstract

In order to fully understand gene function, at some point, it is necessary to study the effects in an intact organism. The creation of the first knockout mouse in the late 1980’s gave rise to a revolution in the field of integrative physiology that continues to this day. There are many complex choices when selecting a strategy for genetic modification, some of which will be touched on in this review, but the principal focus is to highlight the potential problems and pitfalls arising from the interpretation of *in vivo* cardiac phenotypes. As an exemplar, we will scrutinize the field of cardiac energetics and the attempts to understand the role of the creatine kinase (CK) energy buffering and transport system in the intact organism. This story highlights the confounding effects of genetic background, sex, and age, as well as the difficulties in interpreting knockout models in light of promiscuous proteins and metabolic redundancy. It will consider the dose-dependent effects and unintended consequences of transgene overexpression, and the need for experimental rigour in the context of *in vivo* phenotyping techniques. It is intended that this review will not only bring clarity to the field of cardiac energetics, but also aid the non-expert in evaluating and critically assessing data arising from *in vivo* genetic modification.

## Introduction

For over 30 years, scientists have been genetically engineering mouse models in order to study the functional consequences of gain- and loss-of function of a specific protein (Tröder and Zevnik, 2021). This approach revolutionised the study of physiology, since it allowed us to interrogate the effects of very discrete changes at an integrated whole-body level, with all the inherent complexity that this entails. However, the design of such models involves implicit compromises and can be difficult to interpret, at times obfuscating the literature rather than shining a light. Furthermore, for the physiologist, the move towards mice and away from larger, more practicable, sized species proved a steep learning curve and there are many lessons to be learnt.

Herein I will discuss the pitfalls and challenges relating to the *in vivo* assessment of cardiovascular phenotypes in genetic mouse models. By way of example, I have chosen to focus on the myocardial creatine kinase (CK) system, since this aptly demonstrates the principles to be discussed, while also providing a narrative arc. However, the general concepts and themes are widely applicable (Table 1).

**Table 1:** Checklist for planning or evaluating murine *in vivo* phenotypes.

Genetic model
Ubiquitous vs. tissue-specific modification Choice of promoter: determines strength of expression and tissue-specificity Constitutively active vs. inducible Control of integration site and copy number vs. random integration Background genetics
Experimental factors
Blinding and randomisation Choice of anaesthetic Choice of phenotyping methodology Measurement standardisation and validation Power calculations – is the study sufficiently powered to conclude no differences?
Experimental confounders
Are groups matched for sex or analysed separately? Are groups matched for age? Has the genetic background been defined and fixed? Breeding strategy, e.g., use of littermate controls Diet, temperature, light-dark cycle, oestrus cycle, and health status Time of day for experimental measurements Are there whole-body phenotypes? e.g., altered body composition or metabolic status What else does this gene do?

## The Myocardial Creatine Kinase System

**Figure 1:**
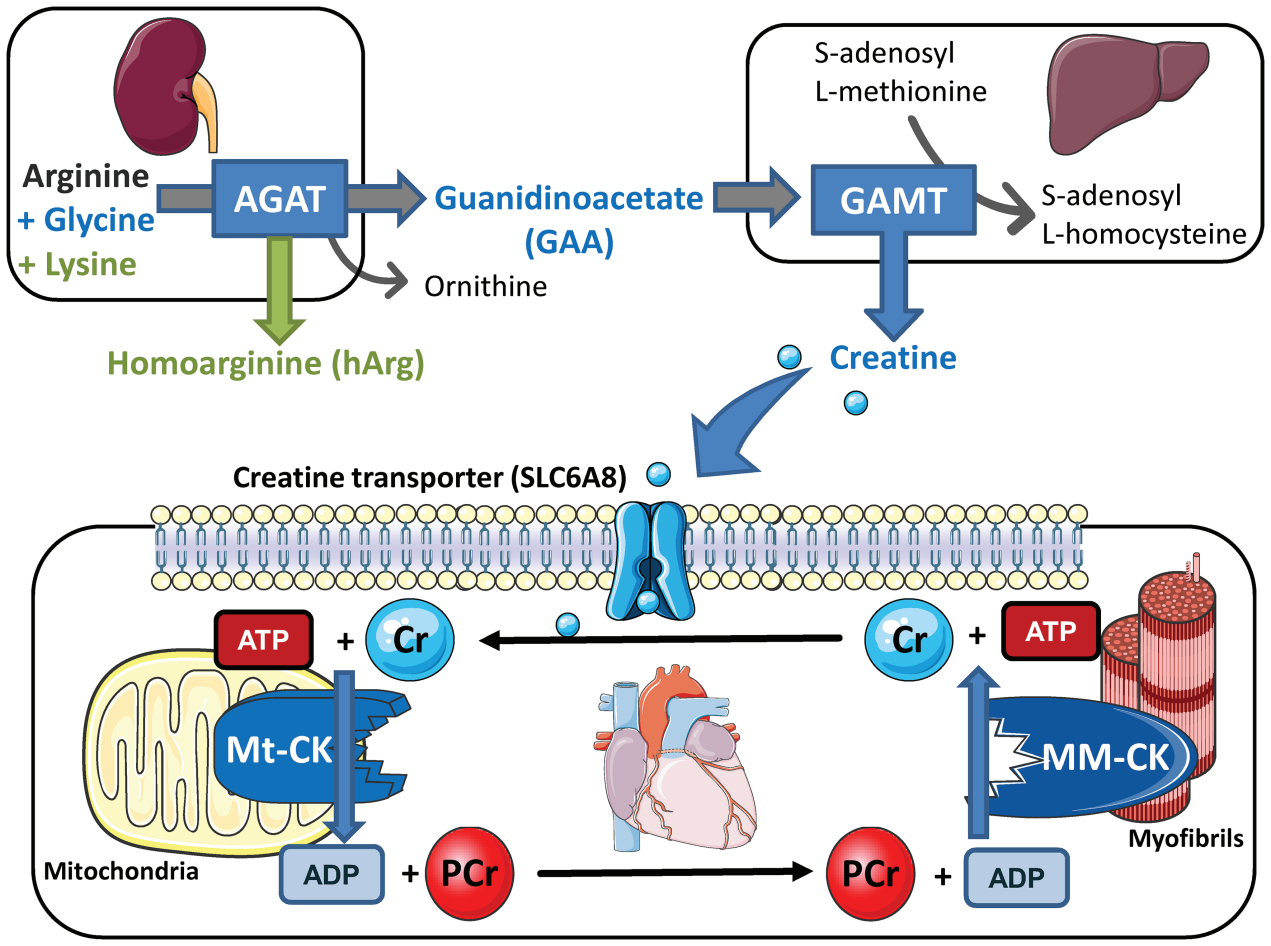
Creatine biosynthesis and the myocardial creatine kinase (CK) phosphagen system. Arginine:glycine amidinotransferase (AGAT; EC 2.1.4.1) is predominantly expressed in the kidneys where it combines arginine and glycine to make guanidinoacetate (GAA) or with lysine to make homoarginine (hArg). Circulating GAA is taken up by the liver where GAA N-methyltransferase (GAMT; EC 2.1.1.2) utilises the methyl group from S-adenosyl L-methionine to synthesise creatine. These biosynthetic enzymes are not expressed in cardiomyocytes so creatine must be taken-up *via* a specific plasma membrane creatine transporter (SLC6A8). Creatine accumulates inside the cell where the sarcomeric mitochondrial isoform of creatine kinase (MtCK; EC:2.7.3.2) catalyses the transfer of a phosphoryl group from ATP to form phosphocreatine (PCr) and ADP. PCr is a high abundance and mobile energy source that can be utilised to rapidly regenerate ATP at times of high demand under the control of the muscle isoform of creatine kinase (the dimer MMCK; EC 2.7.3.2). Free creatine diffuses back to stimulate further oxidative phosphorylation and re-start the cycle. Knockout models exist for all five of these proteins and overexpression models for CK and creatine transporter (CrT). This figure was created using Servier Medical Art by Servier, which is licensed under a Creative Commons Attribution 3.0 Unported License https://smart.servier.com.

Creatine kinase forms part of a cellular energy buffer and transport system that is common to excitable cells in all vertebrates and is summarised in Figure 1 (Wyss and Kaddurah-Daouk, 2000). Within the cardiomyocyte, it facilitates movement of high energy phosphates around the cell; it provides an energy back-up to ensure that ATP levels stay constant at times of high demand; and it buffers local concentrations of ATP and ADP to maintain thermodynamically favourable conditions (Wallimann et al., 1992; Neubauer, 2007). This much we know without resorting to genetically modified mice, principally by using pharmacological tools to inhibit CK activity (e.g., iodoacetamide) or deplete creatine levels (using the creatine-analogue β-guanidinopropionic acid, β-GPA). The former is highly toxic and hence is suitable only for acute *ex vivo* experiments, and the latter competes with creatine for cellular uptake, such that creatine depletion is incomplete and occurs gradually over months (Lygate and Neubauer, 2014). A genetic approach should represent a much cleaner experiment, with greater specificity (no off-target effects) and a more complete disruption of the CK system. It was hoped that this would clearly demonstrate the critical importance of the CK system to normal cardiac function and establish whether the downregulation of the CK system, which is universally observed in the chronically failing heart (Neubauer, 2007), helps to drive pathophysiology.

## Choice of Genetic Expression System

Currently there are a great many choices to be made at the planning stage for a new mouse line, many of which will have a substantial impact on subsequent phenotyping. Traditional constitutive models, i.e., where the gene is modified throughout life, can still represent a reasonable choice since they are relatively simple to generate and work with. The downside is that gene expression is altered during development, which may lead to unintended adverse effects (including embryonic lethality) or the potential for long-term compensatory adaptations that obfuscate the critical nature of the gene being studied. Of course, much depends on the promoter, which can be chosen to provide ubiquitous expression or tissue-specificity, and/or drive weak or strong gene expression (for a review, see Wang, 2009; Doetschman and Azhar, 2012). Consideration should be given as to whether promoter activity is affected by the disease being studied, e.g., the α-MHC gene (*Myh6*) is downregulated in the hypertrophic heart, and it is worth checking empirically whether this is sufficient to alter transgene expression. This and other potential pitfalls are reviewed elsewhere (Molkentin and Robbins, 2009).

All of the CK knockout models are constitutive whole-body knockouts, so the possibility of confounding effects from other tissues and organs has to be considered, e.g., contribution of skeletal muscle and neuroendocrine mechanisms or developmental effects on the brain that might affect the ability to perform phenotyping tasks such as wheel-running.

Conditional or inducible models are a favoured approach to provide both spatial and temporal control, e.g., cardiomyocyte-specific MerCreMer activated by administration of tamoxifen (αMHC-CreER) or the tetracycline-controlled transactivator (tTA) system typically activated by doxycycline (α-MHC-tTA; for review, see Doetschman and Azhar, 2012). The most important consideration from the point-of-view of subsequent phenotyping is the choice of appropriate control groups (Pugach et al., 2015). For example, the Tet system can be “leaky,” i.e., there can be background expression of transgene in the absence of activation by tetracyclines (Doetschman and Azhar, 2012). Chronic treatment with doxycycline alone has been shown to accelerate the progression of left ventricular (LV) hypertrophy and congestive heart failure in a murine model of pressure-overload (Vinet et al., 2008), although others have found a protective effect in a similar model (Errami et al., 2008). Regardless, both these studies urge caution when interpreting disease models in the presence of doxycycline. Similarly, expression of the tTA system has also been shown to protect against ischaemia/reperfusion (I/R) injury *ex vivo* and *in vivo* even in the absence of tetracycline or a transgene payload (Elsherif et al., 2012).

Similar confounders have been described for the αMHC-CreER system, where the combination of Cre expression and high-dose tamoxifen can result in cardiomyocyte apoptosis, fibrosis, and depressed cardiac function even in the absence of a floxed transgene (Koitabashi et al., 2009; Bersell et al., 2013). This cardiomyopathy is reversible and great care should be taken when interpreting phenotypes that emerge in the first 1–2 weeks after tamoxifen administration (Koitabashi et al., 2009). It can also be minimised by reducing Cre expression levels and careful titration of tamoxifen dose to obtain maximal recombination with minimal adverse effects (Bersell et al., 2013). A recent study has shown that the tamoxifen metabolite, 4-hydroxytamoxifen, efficiently induces gene editing without transient or long-term effects on cardiac function, energetics, or remodelling (Heinen et al., 2021). Clearly this represents a promising strategy for optimisation of the αMHC-CreER system.

It is also notable that mice expressing αMHC-Cre alone have been reported to develop cardiac fibrosis and mild LV dysfunction by 6 months of age (Pugach et al., 2015). Taken together, all of the above strongly suggests the need to include controls that express the Cre or tTa constructs (without the transgene) and that are given the activator compound, i.e., wild-type littermates are not an appropriate choice (Bhandary and Robbins, 2015).

Traditional over-expression models result in multiple copies of the transgene with random insertion into the genome. This can cause unintended consequences if the insertion site happens to disrupt another gene that is important for cardiac function (Doetschman and Azhar, 2012). Hence, the necessity to generate at least two transgenic lines and compare the phenotypes before declaring that they arise from manipulation of the transgene. The creatine transporter overexpressing (CrT-OE) mouse is an example of this, which is why there exists two lines from the same lab that have different distributions of myocardial creatine concentrations (Wallis et al., 2005). The technological solution is to specifically insert a single copy of the transgene at the *Rosa26* locus, chosen because of its stable constitutive and ubiquitous expression and the high rate of homologous recombination (Doetschman and Azhar, 2012). This is the approach taken when the mitochondrial creatine kinase overexpressing strain (mito-CK-OE) was created (Whittington et al., 2018), since uncontrolled levels of transgenic protein (e.g., due to multiple gene copies) within the limited real estate of the mitochondrial intermembrane space might be detrimental to the cell. This approach successfully avoided mitochondrial toxicity and provided physiological levels of protein expression; however, with myocardial MtCK activity only elevated by 27%, this also meant larger group sizes were required to detect phenotypic differences (Whittington et al., 2018).

## Experimental Factors

Phenotypes are affected by environmental factors, most of which are controlled by the animal holding facilities, e.g., temperature, humidity, diet, pathogen status, light-dark cycle, companion animals etc. However, it has been well documented that these factors are underreported in the literature, and the ARRIVE guidelines are to be applauded for attempting to correct this oversight (Percie Du Sert et al., 2020). Within the context of the CK system, diet is particularly important since old-style mouse chow includes ingredients of animal origin and will therefore contain creatine. Even relatively small differences in the dietary composition of standard chow can alter outcomes such as gene expression and heart weight (Smeele et al., 2011), which may contribute to variability in findings between different laboratories.

The effect of temperature is often overlooked, but is an important consideration, particularly since recent evidence have shown that creatine in adipocytes has a fundamental role in thermogenesis and controlling adiposity (Kazak et al., 2015, 2019). Mice in general are housed ~10°C below their thermo-neutral point (Hamlin and Altschuld, 2011), so they are expending energy to keep warm, therefore differences between lean and normal mice may compound this effect. Even small changes in ambient temperature have a measurable effect on rodent cardiovascular parameters (Swoap et al., 2004).

Most *in vivo* phenotyping techniques require immobilisation *via* restraint or general anaesthesia and there is no ideal solution. For example, it is possible to perform echocardiography in conscious animals that have been acclimatised to the procedure, but there will always be an element of induced stress to this approach (Lindsey et al., 2018). For general anaesthesia, there is consensus that isoflurane is best for animal welfare due to an ability to titrate doses in real time, rapid recovery, and relatively mild cardiorespiratory depression (Tremoleda et al., 2012). However, on a practical level, there is no good anaesthetic for measuring cardiac function and all have depressant effects to some extent that ultimately has a major impact on measurement variability (Lindsey et al., 2018). Indeed, the ability to “dial” a phenotype based on anaesthetic levels means that the use of blinding and randomisation is critically important in such experiments in order to minimise unconscious bias and/or confirmation bias.

An example of the effect of anaesthetic choice can be seen in early reports using echocardiography in M/Mt-CK^−/−^ (CK double knockout) mice at 6–8 months of age. Impaired contractile reserve was observed compared to C57BL/6 controls under ketamine and xylazine anaesthesia. However, no differences were observed when the experiment was repeated using etomidate anaesthesia, which provided more physiological heart rates (Crozatier et al., 2002). However, it should be noted that with *n* = 5 per group, this study was greatly underpowered to detect a subtle phenotype. The importance of performing power calculations to determine group sizes cannot be underestimated, particularly before concluding there is no phenotype.

## Choice of Phenotyping Techniques

*In vivo* measurements exhibit a high degree of variability, so attempts to standardise methodologies are to be welcomed (Lindsey et al., 2018). Consideration should also be given to standardising training and validating new users against an experienced operator, particularly with echocardiography and MRI since there is a subjective component to the measurements (Donner et al., 2018).

Haemodynamics and imaging focus on different parts of the cardiac cycle and are complimentary rather than interchangeable. For example, we have consistently failed to detect contractile dysfunction using MRI in guanidinoacetate N-methyltransferase (GAMT) and arginine:glycine amidinotransferase (AGAT) knockout mice, yet both exhibit an impairment in LV systolic pressure generation (Ten Hove et al., 2005; Schneider et al., 2008; Faller et al., 2018). Dysfunction may only become apparent under stress conditions (e.g., dobutamine for maximal β-adrenergic stimulation) and this is particularly true when considering cardiac energetics, which are workload-dependent and thought to determine contractile reserve (Kapelko et al., 1988; Tian and Ingwall, 1996).

In theory, LV cannulation to perform pressure-volume (PV) analysis should be the gold-standard phenotyping method, since it informs on all stages of the cardiac cycle and can provide load-independent measures of contractility and relaxation. However, it is intrinsically difficult in the mouse to calibrate absolute volumes from conductance measurements. This usually requires three major corrections: (1) Conversion of conductance to volume units by measuring conductance in wells of known volume; (2) Parallel conductance, i.e., the portion of your signal arising from surrounding tissues rather than blood, which is typically assessed using the hypertonic saline bolus method; and (3) Correction for inhomogeneity of the electrical field (α), which is determined by the interaction with tissue geometry and can lead to large underestimations of stroke volume (Feldman et al., 2000; Jacoby et al., 2006). Thus, stroke volume should ideally be measured by an alternative method (e.g., imaging or flow probe) to determine α correction factor when absolute volumes are required (Nielsen et al., 2007; Clark and Marber, 2013). Furthermore, the surgical preparation can be chest-open or chest-closed. The former involves inserting the PV cannula into the LV *via* the apex and results in lower measured pressures, while the latter is more physiological relevant with access *via* the carotid artery (Lips et al., 2004). Finally, load-independent parameters require alteration of loading conditions, e.g., *via* occlusion of the inferior vena cava. All of the above will affect the fidelity of the data obtained (Pacher et al., 2008).

## Influence of Genetic Background

**Figure 2:**
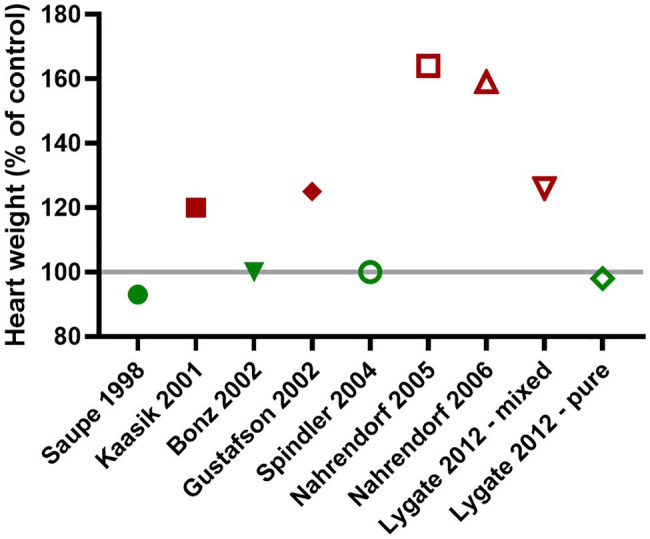
The presence or absence of cardiac hypertrophy in CK double knockout mice (CK-dKO) has varied considerably over time and between laboratories, in large part, due to a mixed and changing genetic background. When finally bred onto a pure C57BL/6 J background, no cardiac hypertrophy was evident (Lygate et al., 2012a,b - pure).

Genetic background is a well-established confounder (Garcia-Menendez et al., 2013), but one that is often ignored since it is expensive and time-consuming to backcross for the >10 generations necessary to be considered congenic (Fontaine and Davis, 2016). A good example of this is in the reported heart weights of CK double knockout mice (CK-dKO), which has been variously reported as normal or grossly hypertrophied, sometimes even from the same laboratory (Figure 2; Saupe et al., 1998; Kaasik et al., 2001; Bonz et al., 2002; Gustafson and Van Beek, 2002; Spindler et al., 2004; Nahrendorf et al., 2005, 2006; Lygate et al., 2012b). This can be explained by the mix of C57BL/6 and 129Sv backgrounds in this strain, with a new mix arising with each generation leading to genetic and phenotypic drift. When my laboratory imported these mice, we phenotyped both the original mixed background and backcrossed onto C57BL/6JOlaHsd. Only on the mixed background did the mice develop LV hypertrophy and contractile dysfunction leading to congestive heart failure, and even then, only in ageing males (Lygate et al., 2012b). Similar experiments in 1-year old Mt-CK^−/−^ on a pure C57BL/6JOlaHsd background did not indicate any cardiac dysfunction at rest or under maximal stimulation (Lygate et al., 2009). Taken together, these studies suggest a threshold minimum, such that only severe CK deficiency can, in itself, cause heart failure, however, it requires a permissive genetic background and depends on sex and age.

From human epidemiology studies, it is no surprise that sex and age have a strong influence on disease severity (Regitz-Zagrosek, 2006), but this is too often ignored and under-reported, with less than 10% of publications providing all of the basic characteristics suggested by the ARRIVE guidelines (Percie Du Sert et al., 2020). There is a big push by many funding organisations to include both males and females in all preclinical studies, but this requires caution when analysing mixed groups to ensure we are comparing like with like.

It is further worth noting that not all C57BL/6 are created equal, with some sub-strains carrying specific mutations that are absent in others (Fontaine and Davis, 2016). The best known of these is the *nnt* gene mutation found in some colonies of C57BL6/J, but not in others (e.g., C57BL6/JOlaHsd) or in C57BL6/N, which can have a profound effect on the development of heart failure (Nickel et al., 2015). Similarly, a loss of function mutation in myosin light chain kinase 3 (*Mylk3*) has been described in C57BL6/N, but not C57BL6/J, which is associated with mild dilated cardiomyopathy at 12 months of age (Williams et al., 2020). These examples highlight the importance of understanding specific sub-strain genetics, which can vary between suppliers, and speaks to the necessity of appropriate littermate controls.

### Creatine-Deficiency and the Law of Unintended Consequences

The GAMT-KO mouse has an absolute whole-body deficiency of creatine, which results in possible confounders of very low fat and body weight (Schmidt et al., 2004). This is unavoidable since GAMT is not expressed in the heart, so a cardiac-specific model would not help here. They also have reduced fertility and are maintained by heterozygous breeding, which means a mix of genotypes in each litter. It quickly became apparent that KO mice could acquire creatine by eating the faeces of wild-type littermates and this necessitates the separation of genotypes shortly after weaning (Schmidt et al., 2004).

Furthermore, normalisation of heart weight to either body weight or tibial length (5% shorter) is inherently misleading, and it is only through histology and gene expression profiling that we can say there is no cardiac hypertrophy in this strain (Ten Hove et al., 2005). There is, however, mild impairment of *in vivo* cardiac function consisting of reduced LV systolic pressure development and blunted contractile reserve (Ten Hove et al., 2005). This may be related to whole-body effects on workload, substrate utilisation, or neuroendocrine mechanisms, since no equivalent phenotype was observed in isolated perfused heart despite an apparent energetic deficit. However, it is equally likely that this reflects the 27% residual creatine levels, since the coprophagia problem had not been resolved at this stage (Schmidt et al., 2004).

A further confounder is that GAMT-KO accumulates high levels of the creatine precursor, GAA, and this can be phosphorylated and utilised in the CK reaction (Ten Hove et al., 2005). We know this gets utilised during ischaemia and that regeneration is very slow (Ten Hove et al., 2005; Lygate et al., 2013), so there could be some compensatory effect, but flux is 100-fold lower than in the presence of creatine (Boehm et al., 1996). Nor can we completely rule-out a potential toxic effect of GAA accumulation to influence the phenotype (Aksentijević et al., 2020).

GAMT-KO is another example of a phenotype that develops with age, and while LV structure does not change (Schneider et al., 2008), we have observed a gradual decline in LV pressure development and heart rate *in vivo* (Aksentijević et al., 2020). However, the absence of pulmonary congestion or elevated LV end-diastolic pressures suggest this is not due to chronic heart failure, but may instead represent an energy-sparing strategy to reduce stroke work (Aksentijević et al., 2020). Perhaps, a sensible strategy for a mouse past the age of reproduction, living in controlled conditions with food and water available *ad libitum*.

It is often argued, and quite reasonably so, that expecting to observe phenotypes in otherwise healthy young mice is unrealistic. We therefore superimposed creatine-deficiency onto a surgical model of ischaemic heart failure. If low creatine levels help drive the phenotype then surely GAMT-KO with zero creatine should do much worse? To our surprise, GAMT-KO hearts did not develop heart failure any more severe than wild-type, despite having mild dysfunction at baseline (Lygate et al., 2013) and this also holds true for CK-dKO mice (Nahrendorf et al., 2005, 2006). This is in stark contrast to rats fed β-GPA in order to reduce creatine levels prior to myocardial infarction (MI), which resulted in 100% mortality at 24 h (Horn et al., 2001), and in a similar study, 93% mortality within 60 min, predominantly due to fatal arrhythmia (Lorentzon et al., 2007). It seems likely that this discrepancy can be explained, at least in part, by species differences, since we previously ruled out off-target effects of β-GPA in mice (Lygate et al., 2013), and rats are much more susceptible to post-MI arrhythmia (Lygate, 2006). It is clearly only an acute effect, since rats fed β-GPA for 8 weeks starting immediately after MI did not exhibit increased mortality and there was no additional haemodynamic impairment, despite lower levels of myocardial creatine and ATP (Horn et al., 2001). This is in good agreement with the findings of our GAMT-KO post-MI study (Lygate et al., 2013); suggesting that further depletion of creatine in the context of chronic heart failure is not detrimental.

The counter-argument is that multiple compensatory adaptations have been observed in CK-KO mice that may explain this mild phenotype, for example, structural reorganisation to reduce the diffusion distance between mitochondria and myofibrils (Kaasik et al., 2001), however, similar adaptations have been sought in GAMT-KO without success (Branovets et al., 2013; Lygate et al., 2013). Nevertheless, this raises the question of multiple metabolic redundancy and whether energy provision in the heart is simply too important to fail. This concept is perhaps best illustrated in yeast where knockouts have been created for every single metabolic gene. This found that only 13% were essential for survival, despite 74% of metabolic genes being involved in processes that were considered essential for growth (Deutscher et al., 2006). This concept extends to complex mammalian biology with 10–15% of mouse knockout strains estimated to have no discernible phenotype suggesting that certain processes are genetically robust (Barbaric et al., 2007). Of course, lack of a phenotype could simply reflect insensitivity of our measurements or that we are using the wrong tests. For example, we could not detect any difference between wild-type and GAMT-KO mice in their ability to perform forced or voluntary running exercise (Lygate et al., 2013), but would that really hold true if faced with real-life predators?

## Agat a Promiscuous Protein

The arrival of AGAT knockout mice was keenly anticipated in the field as a purer model of creatine deficiency since there is no accumulation of GAA, and creatine levels can be precisely controlled *via* dietary intervention (Choe et al., 2013). However, the same potential confounders of whole-body phenotypes persist, for example, severe skeletal muscle atrophy that develops with age (Nabuurs et al., 2013). Notably, these phenotypes are associated with widespread activation of AMP-activated protein kinase (AMPK), suggesting a major energetic deficit, and are completely rescued by dietary creatine (Choe et al., 2013; Nabuurs et al., 2013).

Furthermore, it turns out the AGAT enzyme is a little more promiscuous then previous thought and can also synthesise homoarginine (hArg) from arginine and lysine (Davids et al., 2012; Kleber et al., 2013). Hence AGAT-KO mice have an absolute creatine deficiency and very low hArg levels. Homoarginine has no known metabolic role, but low circulating levels in the plasma have been associated with increased risk of cardiovascular and all-cause mortality in a wide range of human observational studies and have been proposed as a novel biomarker of disease (Atzler et al., 2015; Angelo et al., 2018).

When we characterised the *in vivo* cardiac phenotype in creatine-deficient AGAT-KO mice, we found impairment of LV contractility and relaxation, both at baseline and under maximal β-adrenergic stimulation (Faller et al., 2018). However, none of these parameters were rescued by introducing creatine into the diet despite normalisation of myocardial levels, only low LV end-systolic pressure and heart weight showed improvement, likely due to an osmotic effect. Remarkably, it was hArg supplementation that rescued all other parameters of LV dysfunction, suggesting it is more than just a simple biomarker (Faller et al., 2018), and it might explain why hArg was found to maintain contractile reserve in a murine model of ischaemic heart failure (Atzler et al., 2017).

The consequences of creatine-deficiency are clearly very different between skeletal and cardiac muscle, despite AGAT not being expressed in either tissue. It appears that PCr/ATP levels are maintained in the heart, but are reduced by 46% in skeletal muscle (Nabuurs et al., 2013; Faller et al., 2018). Consequently, only skeletal muscle shows activation of AMPK and downstream signalling, indicating major divergence in metabolic signalling (Nabuurs et al., 2013; Faller et al., 2018).

We can hope that the new models of creatine transporter deficiency (CrT-KO) might bring clarity, especially if made to be cardiac-specific (e.g., by crossing with α-MHC Cre), which will circumvent the confounding whole-body phenotypes. A conditional knockout has indeed been created, but cardiac-specific deletion has yet to be explored (Baroncelli et al., 2014). Two other global knockouts have been reported, but not assessed for cardiac phenotypes. In one, the skeletal muscle phenotype was found to recapitulate the dystrophy observed in AGAT-KO (Stockebrand et al., 2018), while the other has reported unexpectedly high levels of creatine in the heart (22% of control; Skelton et al., 2011). However, the same model assessed in a different laboratory had negligible levels of creatine and creatine uptake in the heart for reasons that are unclear (Wawro et al., 2021).

So where does that leave the role for a functioning CK system? The appeal of a knockout model is the hope of proving causation. If the impairment in CK system observed in heart failure is an important contributor to pathophysiology, then we might expect CK deficiency to recapitulate aspects of a heart failure phenotype. However, as summarised in Table 2, no such clear and unambiguous phenotype has emerged. Whether this is due to compensatory adaptations, metabolic flexibility, or genetic robustness, it amounts to the same thing; having a sub-maximal CK system is compatible with murine cardiac health. This also illustrates the inherent difficulty of working with knockouts; where we have learnt much concerning the underlying biology, but we have not settled the fundamental question of causation.

**Table 2:** Genetically-altered mouse models affecting the creatine kinase system.

Mouse model/Nomenclature	Cardiac functional phenotype	Selected references
	Loss of function	
**MCK-KO** Ckm^tm1Bew^	Limitations: mixed genetic background in early studies No discernible phenotype	Van Deursen et al., 1993; Saupe et al., 1998
**MtCK-KO** Ckmt2^tm1Bew^	Limitations: mixed genetic background in early studies Seldom studied in isolation; typically, as double KO No cardiac dysfunction @ 1 year on C57BL/6 background	Steeghs et al., 1997; Lygate et al., 2009
**CK-dKO** Ckm^tm1Bew^/Ckmt2^tm1Bew^	Limitations: mixed genetic background in early studies Mixed: reduced contractile reserve by echo? Mixed: variable left ventricular (LV) hypertrophy Mixed: LVH and heart failure in males @ 1 year Mixed: normal post-infarct remodelling and dysfunction C57BL/6: no *in vivo* cardiac phenotype @ 1 year	Steeghs et al., 1997; Kaasik et al., 2001; Crozatier et al., 2002; Lygate et al., 2012b
**GAMT-KO** Gamt^tm1Isb^	Limitations: guanidinoacetate accumulation; whole-body phenotype Reduced LV systolic pressure Blunted contractile reserve Increased susceptibility to ischaemia/reperfusion Reduced cardiac stroke work >1 year of age Normal post-MI remodelling and dysfunction Normal forced and voluntary running capacity	Schmidt et al., 2004; Ten Hove et al., 2005; Schneider et al., 2008; Lygate et al., 2013; Aksentijević et al., 2020
**AGAT-KO** Gatm^tm1.1Isb^	Limitations: homoarginine deficiency; severe whole-body phenotype Reduced LV systolic pressure (rescued by creatine) Low heart weight (rescued by creatine) Impaired contractility and relaxation (rescued by hArg) Impaired contractile reserve (rescued by hArg)	Choe et al., 2013; Faller et al., 2018
**CrT-KO** Slc6a8^−/y^ B6(Cg)Slc6a8^tm1.2Clar^/J	Limitations: whole-body phenotype; residual creatine? Low heart weight; *in vivo* heart phenotype not determined	Skelton et al., 2011; Wawro et al., 2021
	Gain of function	
**MCK-OE** Tg(Myh6-Ckm)	Limitations: caution with controls and doxycycline dosing No discernible phenotype at baseline Improved recovery from ischaemia/reperfusion Protects against doxorubicin cardiotoxicity Protects against pressure-overload induced chronic heart failure by maintaining myocardial energetics	Akki et al., 2012; Gupta et al., 2012, 2013
**MtCK-OE** Rosa26^tm1(Myh6-Ckmt2)^	Limitations: mild overexpression of transgene No discernible phenotype at baseline Protects against ischaemia/reperfusion injury, but not chronic heart failure despite maintaining normal PCr/ATP	Whittington et al., 2018; Cao et al., 2020
**CrT-OE** Tg(Myl2-Slc6a8)-55	Limitations: highly variable creatine levels Very high levels of myocardial creatine cause heart failure Moderate creatine elevation protects against ischaemia/reperfusion injury but not chronic heart failure	Wallis et al., 2005; Phillips et al., 2010; Lygate et al., 2012a

## Gain-of-Function Models

Just because deficiency of a specific protein is not disease causing *per se*, does not infer that augmenting that protein cannot positively influence outcomes in relevant disease models. The best example of this is for over-expression of M-CK, where the knockouts have no discernible functional heart phenotype (Saupe et al., 1998), yet specific overexpression in the heart was found to protect against LV dysfunction and improved survival in a pressure-overload model of chronic heart failure (Gupta et al., 2012). MCK-OE mice were created using the tTA “Tet-off” system to elegantly show cause and effect. In this configuration, administration of doxycycline supresses the transgene, which is only activated when doxycycline is removed from the diet. This allowed the transgene to be switched-off mid-way through the experiment in a subset of transgenic mice, whose energetic status and cardiac function then regressed (Gupta et al., 2012). This is the first demonstration that augmentation of the CK system can protect against chronic heart failure. It represents particularly strong evidence, since the transgene is only switched on during adulthood, thereby circumventing developmental adaptations, but with the caveats that the genetics of the control group are poorly defined and we are comparing mice with and without the potentially confounding effects of doxycycline administration. Cardioprotection by MCK-OE has since been demonstrated in both doxorubicin cardiotoxicity and in an I/R model (Akki et al., 2012; Gupta et al., 2013).

My laboratory has created a constitutive cardiac-specific Mt-CK overexpressing strain and subjected it to the same pressure-overload model. While we observed a relative preservation of PCr/ATP levels in the failing hearts, there was no impact of Mt-CK expression on LV remodelling or *in vivo* dysfunction, indicating that improved energetics does not always mean improved function (Cao et al., 2020). Since activity of both M-CK and Mt-CK is reduced in the failing heart, this suggests that the flux of energy to the myofilaments is more limiting to contractile function than is the generation of PCr by mitochondria. It also suggests the possibility that overexpressing both CK isoforms might be synergistically beneficial in chronic heart failure. However, in the setting of acute ischaemia, it should be noted that Mt-CK overexpression strongly protected against I/R injury *in vivo* and improved functional recovery *ex vivo* (Whittington et al., 2018).

One major issue with overexpressing proteins is the potential for unregulated supra-physiological expression of gene products that may even be toxic to the cell. My laboratory created a model of cardiac-specific CrT overexpression in order to force more creatine into cardiomyocytes (Wallis et al., 2005). For reasons that are not clear, this resulted in very large individual variations in myocardial creatine levels, with levels above 2-fold associated with LV hypertrophy and dysfunction due to impaired glycolysis and an inability to maintain the ratio of creatine to phosphocreatine (PCr; Wallis et al., 2005; Phillips et al., 2010). Hence, combining this model with CK overexpression may prove informative. We have since defined a safe level of “moderate” creatine elevation in the heart (+20–100%) and shown that this protects against I/R injury, however, it was not effective in an ischaemic model of chronic heart failure (Lygate et al., 2012a).

Overall, the evidence from GA mouse studies is unequivocal for I/R injury. An impaired CK system is consistently detrimental, while overexpression of either MCK, MtCK, or CrT is cardioprotective. In the context of chronic heart failure, the knockout models suggest that impairment of the CK system does not drive pathophysiology, but that targeted MCK overexpression may still be beneficial.

## Conclusion

This review highlights some of the confounding variables that are important in the interpretation of *in vivo* cardiac phenotypes in genetic mouse models. Potential pitfalls have been illustrated by examples from the CK system literature, but this is not unique, and no doubt a similar approach could be taken for any biological system. The knockout models have demonstrated that there is little evidence that impairments in the CK system, as observed in the chronically failing heart, are causative or drive the disease process. Notwithstanding, augmentation of some aspects of the CK system would appear to hold therapeutic potential for both I/R injury and chronic heart failure and require further study.

## Author Contributions

The author confirms being the sole contributor of this work and has approved it for publication.

### Conflict of Interest

The author declares that the research was conducted in the absence of any commercial or financial relationships that could be construed as a potential conflict of interest.
